# Foliar Spraying with ZnSO_4_ or ZnO of *Vitis vinifera* cv. Syrah Increases the Synthesis of Photoassimilates and Favors Winemaking

**DOI:** 10.3390/plants13141962

**Published:** 2024-07-17

**Authors:** Diana Daccak, Ana Coelho Marques, Cláudia Campos Pessoa, Ana Rita F. Coelho, Inês Carmo Luís, Graça Brito, José Carlos Kullberg, José C. Ramalho, Ana Paula Rodrigues, Paula Scotti-Campos, Isabel P. Pais, José N. Semedo, Maria Manuela Silva, Paulo Legoinha, Carlos Galhano, Manuela Simões, Fernando H. Reboredo, Fernando C. Lidon

**Affiliations:** 1 Departamento de Ciências da Terra, Faculdade de Ciências e Tecnologia, Campus da Caparica, Universidade Nova de Lisboa, 2829-516 Caparica, Portugal; amc.marques@campus.fct.unl.pt (A.C.M.); c.pessoa@alumni.fct.unl.pt (C.C.P.); arf.coelho@campus.fct.unl.pt (A.R.F.C.); idc.rodrigues@campus.fct.unl.pt (I.C.L.); mgb@fct.unl.pt (G.B.); jck@fct.unl.pt (J.C.K.); mma.silva@fct.unl.pt (M.M.S.); pal@fct.unl.pt (P.L.); acag@fct.unl.pt (C.G.); mmsr@fct.unl.pt (M.S.); fhr@fct.unl.pt (F.H.R.); fjl@fct.unl.pt (F.C.L.); 2 Centro de Investigação de Geobiociências, Geoengenharias e Geotecnologias (GeoBioTec), Faculdade de Ciências e Tecnologia, Campus da Caparica, Universidade Nova de Lisboa, 2829-516 Caparica, Portugal; cochichor@mail.telepac.pt (J.C.R.); paula.scotti@iniav.pt (P.S.-C.); isabel.pais@iniav.pt (I.P.P.); jose.semedo@iniav.pt (J.N.S.); 3 Plant Stress & Biodiversity Lab, Centro de Estudos Florestais (CEF), Laboratório Associado TERRA, Instituto Superior Agronomia (ISA), Universidade de Lisboa (ULisboa), Quinta do Marquês, Avenida da República, 2784-505 Oeiras, Portugal; 4 Plant Stress & Biodiversity Lab, Centro de Estudos Florestais (CEF), Laboratório Associado TERRA, Instituto Superior Agronomia (ISA), Universidade de Lisboa (ULisboa), Tapada da Ajuda, 1349-017 Lisboa, Portugal; anadr@isa.ulisboa.pt; 5 Instituto Nacional de Investigação Agrária e Veterinária, I.P. (INIAV), Avenida da República, Quinta do Marquês, 2780-157 Oeiras, Portugal

**Keywords:** chlorophyll *a* fluorescence, leaf gas exchange, Syrah, total soluble sugars (°Brix), Zn agronomic biofortification

## Abstract

Zinc enrichment of edible food products, through the soil and/or foliar application of fertilizers, is a strategy that can increase the contents of some nutrients, namely Zn. In this context, a workflow for agronomic enrichment with zinc was carried out on irrigated *Vitis vinifera* cv. Syrah, aiming to evaluate the mobilization of photoassimilates to the winegrapes and the consequences of this for winemaking. During three productive cycles, foliar applications were performed with ZnSO_4_ or ZnO, at concentrations ranging between 150 and 1350 g.ha^−1^. The normal vegetation index as well as some photosynthetic parameters indicated that the threshold of Zn toxicity was not reached; it is even worth noting that with ZnSO_4_, a significant increase in several cases was observed in net photosynthesis (P_n_). At harvest, Zn biofortification reached a 1.2 to 2.3-fold increase with ZnSO_4_ and ZnO, respectively (being significant relative to the control, in two consecutive years, with ZnO at a concentration of 1350 g.ha^−1^). Total soluble sugars revealed higher values with grapes submitted to ZnSO_4_ and ZnO foliar applications, which can be advantageous for winemaking. It was concluded that foliar spraying was efficient with ZnO and ZnSO_4_, showing potential benefits for wine quality without evidencing negative impacts.

## 1. Introduction

Zinc is one essential micronutrient in plants implicated in the maintenance of the structural and functional integrity of biological membranes, protein synthesis, and gene expression and regulation [1]. In this context, it was reported that the optimal level of Zn can vary between 20 and 60 mg.kg^−1^_DW_; however, depending on the plant genotype, soil type, and climate, plant species can display different uptake rates from soil, as well as different translocation ratios to the shoots and sequestration depositions in the plant organs [2,3,4]. Worldwide Zn deficiency, mainly in sandy soils, affects the yield, productivity, and quality of several agronomic crops (namely the vigor of germination and biomass), promoting deficiency symptoms, such as stunted growth, chlorosis, and smaller leaves, as well as susceptibility to injury [5,6,7]. Additionally, although varying with plant species, these effects are closely linked to metabolic inhibition, namely decreased levels of phytohormones [8] and the inhibition of cell division, enlargement and differentiation [1], photosynthesis [9], and sterility [10]. To mitigate this problem, several approaches are being considered, including Zn enrichment through foliar applications, to increase the Zn contents in crop plants [11,12,13,14]. In fact, foliar application avoids the fixation and immobilization of nutrients in soils and seems to have higher efficiency in relation to their translocation to the shoots during the productive cycle [15,16,17]. Besides this, Zn translocation in plants, as Zn^2+^ or bound to organic acids, due to a higher concentration of solutes occurs through remobilization in the xylem and mostly through the phloem [18]. Moreover, Zn toxicity, dependent on plant species, might occur between 300 and 400 mg.kg^−1^_DW_ and can affect plant development, namely through reduced growth, the degradation of pigments, the disruption of enzymatic activities, and the inhibition of photosynthesis (in most cases limiting Rubisco activity and photosystem II functioning) in close association with a decrease in the foliar intercellular CO_2_ concentration and stomata conductance [19,20].

Under different climatic conditions, namely temperature and water availability, vines, despite being a resistant crop, face challenges to develop quality attributes and to maximize their winemaking potential [21,22]. For instance, the high acidity, mostly provided by malic and tartaric acids, that characterizes wine grapes balances the flavor profile and affects the wine’s aging potential, whereas foliar Zn application increases the sugar content. Following agronomic approaches, it is therefore important to study, under irrigation, the use of fertilizers to optimize grape quality [23]. Accordingly, using as a test system the genotype *Vitis vinifera* L. cv. Syrah through foliar spraying with ZnSO_4_ and ZnO, this study aimed to develop a workflow for the Zn enrichment of grapes, further considering the physiological response of the vines and the impacts on the quality attributes of wine.

## 2. Results

**Figure 1:**
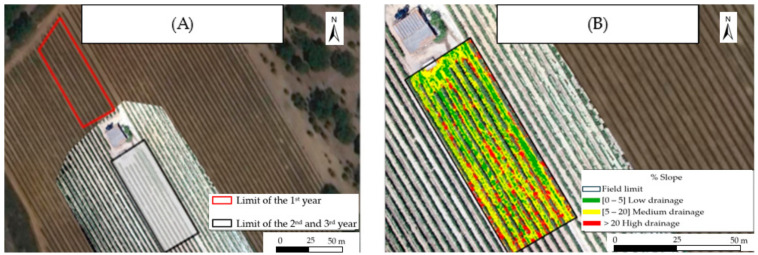
(**A**) Orthophotomap of the 1st year (outlined with a red line) and 2nd and 3rd year (outlined with a black line) (**B**) digital map of slopes of the vineyard of *Vitis vinifera* cv. Syrah grapes.

Field orography characterized through images acquired using a UAV (Unmanned Aerial Vehicle) allowed the definition of slopes and, concomitantly, the estimation [24] of the surface water drainage capacity of the different superficial drainage areas (Figure 1). It was found that, in the vineyard, about 35% of the area could promote the accumulation and/or infiltration of surface water, whereas the remaining 65% corresponds to the aptitude for surface drainage (Figure 1B).

**Figure 2:**
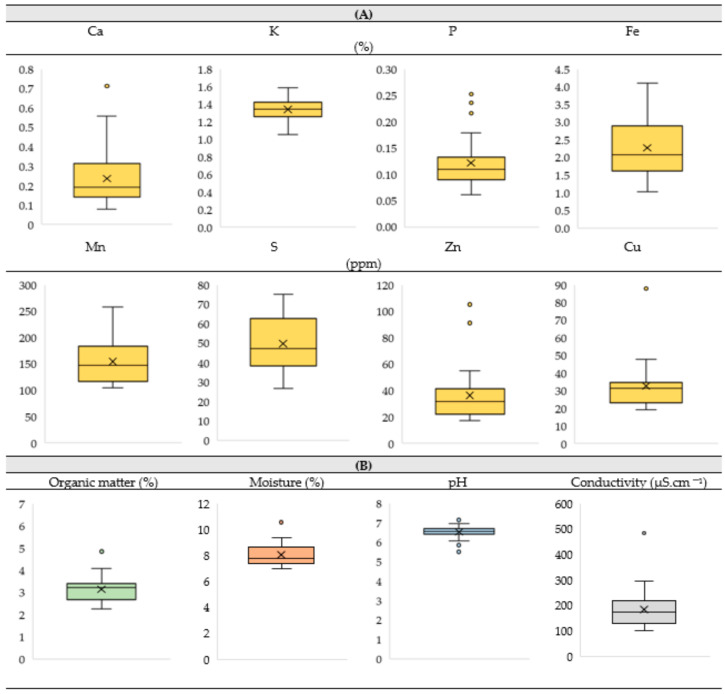
Soil characterization (at about 30 cm depth) in the vineyard. (**A**) Mineral elements (Ca, K, P, and Fe (in %) and Mn, S, Zn, and Cu (in ppm)); (**B**) organic matter (%), moisture (%), pH, and conductivity (µS.cm^−1^) of the soil.

The mineral content of the soil of the vineyard revealed higher levels of Ca, K, P, and Fe (i.e., 0.06–4.09%) and lower amounts of Mn, S, Zn, and Cu (i.e., 17.2–257.7 ppm) (Figure 2A). Moreover, the soil showed average values of 3.14%, 8.04%, and 186 µS.cm^−1^ for organic matter, moisture, and electrical conductivity, respectively (Figure 2B). The pH was slightly acidic, displaying an average value of 6.5 (Figure 2B).

**Figure 3:**
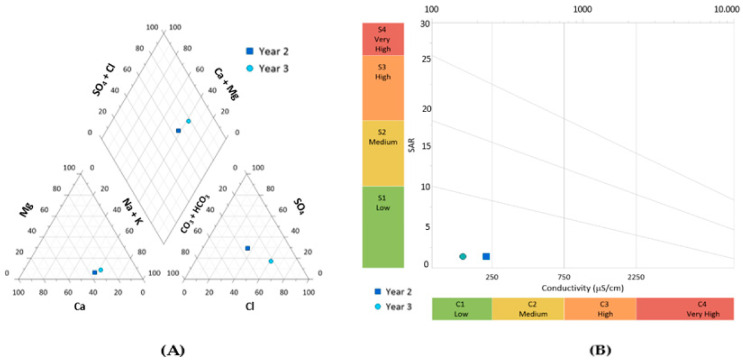
Physicochemical characterization of irrigation water in the vineyard of Syrah during the 2nd and 3rd years of the experimental period. Projection of water sample with (**A**) a ternary Piper diagram; (**B**) a Wilcox diagram.

The irrigation water of Syrah was of underground origin (displaying, in the second and third years, hydrochemic sodium sulfate–chloride and sodium chloride facies, respectively) with low salinity (concentration of salts evaluated in terms of electrical conductivity: between 100 and 250 μS.cm^−1^ at 20 °C) (Figure 3). The Syrah irrigation water belonged to class C1S1, with SAR index 1.4 (Figure 3B).

**Figure 4:**
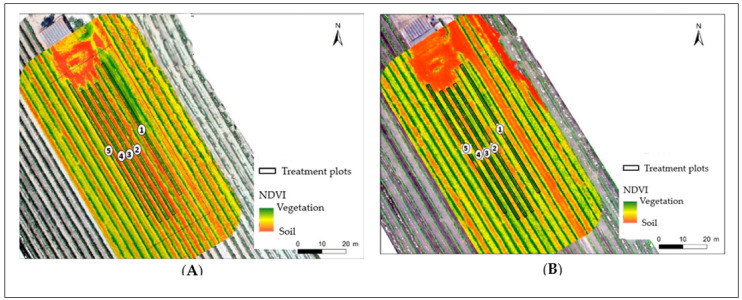
Normalized difference vegetation index (NDVI) of the vineyard with *Vitis vinifera* cv. Syrah during the experimental period (2nd and 3rd year, after the 4th and 1st foliar applications with ZnO or ZnSO_4_, respectively). Syrah: 1—control; 2—ZnO (900 g.ha^−1^); 3—ZnO (1350 g.ha^−1^); 4—ZnSO_4_ (900 g.ha^−1^); 5—ZnSO_4_ (1350 g.ha^−1^). (**A**) Second year of the experimental period (rows 1 to 5 were used); (**B**) third year of the experimental period (the 2nd and 4th rows were not used).

In the second year of the experimental period, and after the fourth foliar spraying, the NDVI of the vineyard showed higher values in the control and 900 g.ha^−1^ ZnSO_4_ treatment groups (Figure 4; Table 1). In the third year, after the first application, vines treated with the ZnO and ZnSO_4_ treatments exhibited (Figure 4; Table 1) a higher vigor (with a maximum value of 0.89, with ZnSO_4_ at 1350 g. ha^−1^).

Considering the second and third years of the experimental period, the vines’ average vigor ranged between 0.55 and 0.66 and from 0.60 to 0.65, respectively. Still, in these cases, the vigor of the vines remained similar with the predominance of green areas (Figure 4; Table 1).

Following the workflow of Zn enrichment in the vines, at a physiological level, significant differences among the treatments and assessment dates of each year were found. Considering the leaf rates of net photosynthesis (P_n_), on the first assessment date, significant differences could not be found among treatments (except in the third year, where the treatment with ZnO at 1350 g.ha^−1^ revealed a lower value relative to ZnSO_4_). Following the second assessment, relative to the control, significantly higher values were found in the first and second years with treatment ZnSO_4_ at 900 g.ha^−1^ and with both treatments at the higher concentrations (1350 g.ha^−1^), respectively (Table 2). Additionally, a significant decrease between the first and second assessment dates among the three years was observed (except in the first year with the ZnO treatment at 900 g.ha^−1^, and in the second year with the leaves sprayed with 1350 g.ha^−1^) (Table 2). Concerning the stomatal conductance to water vapor parameter (g_s_), we found an absence of significant differences between treatments among the last two years of the experimental period (with the first year having a higher value for the ZnO treatment at 900 g.ha^−1^ on the second assessment date) (Table 2). Between both assessment dates of each year, the same pattern was observed with no significant differences in the last two years, with a significant increase being found for ZnO at 450 g.ha^−1^ and ZnO at 900 g.ha^−1^ in the first year (Table 2). Considering the transpiration parameter (E), it was found that the first assessment date of each year did not reveal significant differences between treatments (except in the first year, as ZnO at 900 g.ha^−1^ showed a significantly lower value relative to the control). On the second assessment date, among the treatments, we further found (Table 2) significantly higher values for the control and with treatment ZnSO_4_ at 900 g.ha^−1^ (relative to ZnSO_4_ at 450 g.ha^−1^ and ZnO, in the first year) and with treatment ZnSO_4_ at 1350 g.ha^−1^ (relative to the control and ZnSO_4_ at 900 g.ha^−1^, in the second year). In the first year of the experimental period, and between both assessment dates, the transpiration rates (E) did not display significant differences (Table 2) between the ZnO-treated vines at 900 g.ha^−1^ and the vines treated with ZnSO_4_ at 450 g.ha^−1^. Moreover, in the second and third years, significant differences could not be found in the transpiration rates (except in the control of the third year). Relative to the leaf instantaneous water use efficiency (iWUE), significant differences could not be found among treatments in the second year of the experimental period (Table 2). In the first and third year, the control samples revealed (Table 2) a significantly lower value relative to the other treatments (except in the second assessment of the first year, relative to the treatment with ZnSO_4_ at 450 g.ha^−1^; and in the first assessment of the third year, where the values for ZnO-treated samples remained significantly lower relative to those achieved with the other treatments; and in the second assessment, where spraying the samples with ZnSO_4_ was the only treatment that achieved significantly higher values). Considering the three years, between the two assessment dates of each year, there was a significant decrease between the first and second assessments (Table 2).

The maximum efficiency of PSII (F_v_/F_m_) and the actual PSII photochemical efficiency chlorophyll parameter (F_v_′/F_m_′) showed no relevant differences between the treatments and assessment dates in each year of the experimental period (except in the third year, where an increase between the assessment dates was found for F_v_/F_m_ with treatment ZnSO_4_ at 1350 g.ha^−1^), revealing the maintenance of a high efficiency (Table 3). Concerning the set of estimates of the quantum transport yields, the photosynthetic non-cyclic electron transport (Y_(II)_) did not show relevant variations among treatments in the first and third years. Moreover, on the second assessment date of the second year with ZnSO_4_ at 1350 g.ha^−1^, a significant increase was found (Table 3). In addition, between both assessment dates, only in the third year was a significant decrease found in the control samples (Table 3). As for the regulated energy dissipation (Y_(NPQ)_) and the unregulated dissipation (heat and fluorescence—Y(_NO_)), no significant variations were detected among treatments (except in the second assessment of the second year, where Y(_NPQ_) with ZnSO_4_ at 1350 g.ha^−1^ showed a significantly lower value relative to the control) (Table 3). Additionally, between assessment dates, only the third year showed (Table 3) a significant decrease in all of the samples (except the parameter Y(_NO_), with ZnSO_4_ at 1350 g.ha^−1^, as well as (Y(_NPQ_)) with the control and ZnO at 1350 g.ha^−1^, which increased). Regarding the proportion of energy dissipated as heat through photoprotective mechanisms (q_N_) and energy captured by the PSII open reaction centers and used for photochemical events (q_L_), significant differences were also not found among treatments (Table 3) relative to the control and assessment dates (except the q_N_ parameter in the third year, where only ZnSO_4_ at 1350 g. ha^−1^ did not decrease). Thus, foliar spraying with Zn at all concentrations (i.e., 450, 900, and 1350 g. ha^−1^ of ZnO and ZnSO_4_) kept the performance of the photosynthetic machinery stable and showed a potential positive effect (Table 3).

In the first year of the experimental period, the implications of foliar spraying with ZnO and ZnSO_4_ during the productive stage of the vines determined a non-significant increase in Zn in the grapes at harvest (Table 4). Also, in the second year, the same was observed at harvest (Table 4), but with ZnO at 1350 g.ha^−1^ showing significantly higher values relative to the control (i.e., a 2.3-fold increase). In the last experimental year (after the fourth foliar application at harvest), the results showed (Table 4) a similar trend (i.e., as detected in the first and second years), with a significant increase achieved with ZnO at 1350 g.ha^−1^ relative to the control (i.e., a 1.2-fold increase).

**Figure 5:**
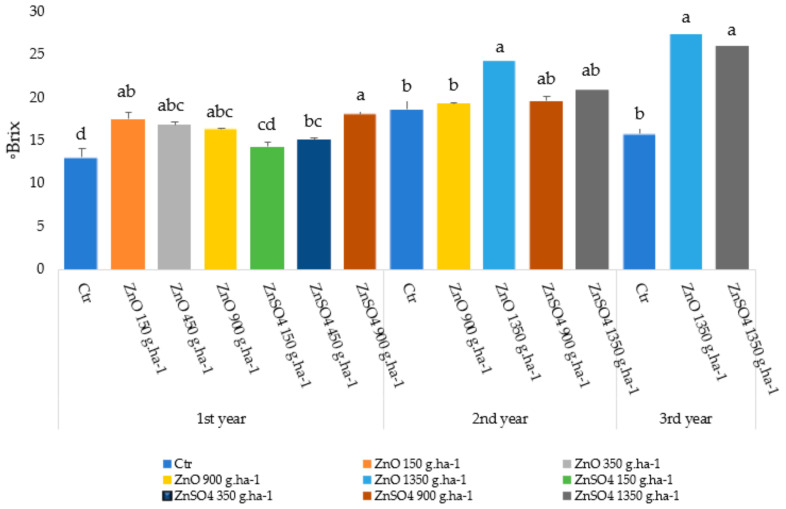
Average ± S.E (*n* = 3) total soluble solids (expressed in ^o^Brix) in grapes of *Vitis vinifera* variety Syrah at harvest during the 1st, 2nd, and 3rd years of the productive cycle. Letters a, b, and c, d indicate significant differences among the treatments (statistical analysis using the one-way ANOVA test, *p* ≤ 0.05).

At harvest, the total soluble solids in the grapes showed higher levels with Zn treatments in each year of the experimental period (Figure 5). In the first year, all of the samples showed significantly higher levels than the control grapes (except ZnSO_4_ at 150 g.ha^−1^) and the values ranged between 13.1 and 17.5 °Brix (Figure 5). In the second year, a significant higher value was found with ZnO at 1350 g ha^−1^ and the values varied between 18.7 and 24.3 °Brix (Figure 5). In the third year, Zn spraying with both treatments at 1350 g.ha^−1^ led to a significant increase relative to the control, and the values ranged between 15.8 and 27.4 °Brix (Figure 5).

## 3. Discussion

The efficiency of Zn enrichment through foliar spraying in the edible part of a plant species is dependent on edaphoclimatic conditions. Considering that over the 3 years of the experimental period, the vineyard was characterized by minimum and maximum temperatures and air humidity ranging between 16.6 and 28.2 °C and from 38 to 90%, the photosynthesis of the vines occurred under favorable environmental conditions and without negative impacts on the vegetative development. In addition, since the soil is the main source of Zn uptake, an adequate amount of available water is required to meet the nutrient needs of crops. In this context, the aptitude of the surface drainage of the vineyard (65%) avoided excess water accumulation and the consequent radicular anoxia in the roots of the vines, as the rise in the water level could replace the oxygen in the soil [25]. Additionally, as the pH of the soil determines the uptake rates of Zn, the vineyard further showed (Figure 2B) an acceptable value (i.e., 6.5, which is in the acceptable range of 5.5–8.5 for this parameter) [26,27]. The organic matter and electrical conductivity of the vineyard for adequate growth also remained close to an adequate range (i.e., 3.14% and 186 µS.cm^−1^—Figure 2B) [28,29,30], therefore being the low electric conductivity associated with a shorter energy expenditure for water uptake by roots [31,32]. In this framework, the moisture of the soil until the 30 cm layer, although susceptible to variations due to precipitation and the evaporation of water of the topsoil [33], revealed (Figure 2B) a value below 10% near the soil surface (15 cm layer). Still, irrigation that ensures soil moisture is a fundamental strategy for preventing water stress at different growth stages, especially in the Mediterranean climatic conditions prevailing in the studied vineyard. Indeed, the water requirements for the physiological functioning (transpiration, osmotic potential, turgor potential, and photosynthesis) of vines, which might affect the productivity and quality of their grapes [34,35], are expected to augment in the near future, with climate projections pointing to an increase in vine irrigation between 3.5 and 7.5% in arid and semi-arid regions [34,36]. Nevertheless, the water quality of the studied vineyard did not show any restriction for agricultural use, being suitable without danger of soil salinization (i.e., 100–250 μS.cm^−1^ of electric conductivity) and alkalinization (i.e., a 1.4 SAR value). In addition, the mineral composition of the soil of the vineyard was further found to be suitable for maintaining the nutritional balance of the vines (Figure 2A), as Fe, K, Ca, P, Mn, and S remained within the ranges of 0.5–4.0%, 0.2–3.0%, 0.2–1.5%, 0.01–0.1%, 40 –850 ppm, and 30–400 ppm, respectively [37,38,39], whereas the average values of Zn and Cu remained in the expected intervals reported for natural soils, i.e., 5–80 ppm and 20–300 ppm, respectively [40].

The monitoring of the vine status was performed through the normalized difference vegetation index (NDVI) in relation to leaf area, fruit ripening and maturity, diseases, water stress, the anthocyanin content of the grapes, and the tannins in their skin [41]. In this context, the Syrah genotype in the vineyard showed an average NDVI varying between 0.55 and 0.65 (Figure 4; Table 1), corresponding to areas with dense vegetation (i.e., positive NDVI values and values above 0.5) and indicating a suitable productive cycle of the plants with minimal stress effects. In fact, similar to data obtained by other authors [42,43], whose proximal-based NDVI values ranged from 0.60 to 0.66, the values found for this vineyard seem to indicate that the amount of anthocyanin in Syrah grapes can be advantageous for achieving greater quality in winemaking [44].

Considering the favorable edaphoclimatic conditions of the vineyard, as also shown by the NDVI results, leaf spraying with ZnO and ZnSO_4_ further stimulated the functioning of the photosynthetic apparatus and even showed a significant increase on the second assessment date of the first and second year (with ZnSO_4_ at 900 g.ha^−1^ and with all treatments at the higher dose of 1350 g.ha^−1^) (Table 2). As previously reported [45], this interaction further suggests that an increase in Zn content promotes the catalytic activity of carbonic anhydrase, facilitating the diffusion of CO_2_ into the chloroplasts and the regulation of HCO_3_ and K^+^ uptake by the guard cells that control stomatal opening. Yet, some treatments on the vines showed a small impact on P_n_ (with only ZnO in the third year at the highest concentration being significant compared to the control), linked to a lower stomatal aperture (g_s_) (Table 2). Likewise, when the threshold of Zn toxicity is reached, P_n_ and E can decrease without visible symptoms due to an anormal functioning of physiological and chemical processes [46]. However, our data indicated that the threshold of Zn toxicity was not reached (as also expressed by our NDVI data), but some other factors, such as (on a daily basis) high temperatures and low relative humidity, eventually, to some extent, inhibited photosynthesis through the gradual closure of stomata to prevent water loss through transpiration [47,48,49]. Indeed, the iWUE was maintained in most cases and also improved significantly in the first year for all treatments (except for ZnSO_4_ at 450 g.ha^−1^) and in the third year with ZnSO_4_ (Table 2). Similar results have been observed in chickpea and wheat plants [50], where foliar application of Zn increased the iWUE through the activation of metabolic processes and osmotic regulation. Complementarily, chlorophyll *a* parameters also indicated that the threshold of Zn toxicity was not reached since F_v_/F_m_, Y(_NO_), Y(_II_), Y (_NPQ_), q_L_, and q_N_ did not show significant changes (Table 3). Although the maximum efficiency of PSII (F_v_/F_m_) showed some sensitivity to the application of the maximum doses of both products applied (1350 g.ha^−1^), there was no impact on the actual photochemical efficiency (F_v_′/F_m_′) (Table 3). Also, relative to the control (Table 3), there was no impact on the parameters that reflect photochemical performance (Y_(II)_, q_L_), the need for energy dissipation processes (Y(_NPQ_), q_N_), or the need for unregulated energy dissipation processes (Y(_NO_)). Thus, all of these parameters, closely implicating PSII, further indicated the absence of relevant inhibitory effects on photosynthesis [51]. As a matter of fact, in the second year, there was even an increase in ZnSO_4_ with the 1350 g.ha^−1^ treatment (at the second assessment, and in the parameters that evaluate photochemical performance) (Y(_II_), q_L_) (Table 3). Considering that the photoprotective mechanism is most active in the early stages, decreasing as the fruit develops [52,53], these data suggested that the functioning of the photosynthetic machinery is extended in the final stage of the vine’s life cycle. Additionally, in this case, we observed a decrease in the need for energy dissipation processes (Y(_NPQ_), q_N_), and no change in unregulated energy dissipation processes (Y(_NO_)), in comparison to the control (Table 3).

The interaction between leaf spraying with ZnO or ZnSO_4_ and the synthesis of photoassimilates promoted (Table 4) Zn accumulation in the grapes (significantly with ZnO at the maximum concentration applied—1350 g.ha^−1^—in the second and third years of the experiment). Nevertheless, it must be noticed that in spite of the lower solubility of ZnO, both Zn sources seem to have similar bioavailability in plants [54,55,56,57], but their efficiency might vary since they are related to different pathways that affect the rate of Zn translocation through phloem to the other tissues [56]. In fact, relative to the soil efficiency, foliar application of Zn is more effective [58,59] and can, to some extent, determine the growth stage of plant development [59,60]. In this context, the total soluble sugars in the mature grapes under the different Zn treatments remained in the range of 13.1 to 27.4 °Brix (Figure 5), therefore persisting within the usual range previously found [61] in other non-treated Zn samples (i.e., usually varying between 13.7 and 31.5 °Brix). As it is known, in winemaking, the sugar content in grapes determines the alcohol concentration, the synthesis of organic acids, the phenolics, and the aroma compounds, which are responsible for sensory properties. Likewise, considering that 22 to 28 °Brix can lead to a greater quality of wine [55,56], similar values were found in the grapes (Figure 5) submitted to both treatments at the higher concentration (i.e., 1350 g. ha^−1^).

## 4. Materials and Methods

The workflow for Zn enrichment of wine grapes was carried out on irrigated *Vitis vinifera* cv. Syrah (having the 1103P rootstock), in a vineyard located in Palmela, Portugal (GPS coordinates: in the first year—38°35′23.629″ N; 8°51′46.208″ W, and in the following two years—38°35′20.84975562″ N; 8°51′43.39046267″ W). After flowering, the aerial part of the vine plants was pulverized with ZnSO_4_ or ZnO (except the control plants, which were sprayed with water) at concentrations of 150, 450, 900 g.ha^−1^ (in the 1st year); 900 and 1350 g.ha^−1^ (in the 2nd year); and 1350 g.ha^−1^ (in the 3rd year). Three and four foliar applications (in the first and latest two years, respectively) were performed. The intervals of pulverizations (from the 1st application to harvest) was about 15 days.

Considering the intervals of foliar spraying (from the first application until harvest) for each year, data on the temperature and air humidity were assessed in the national meteorological networks of Montijo, Portugal (38°43′28.7″ N; 9°0′40.114″ W) and Setubal, Portugal (38°31′12″ N; 8°52′48″ E) for the first year and thereafter for the following years of the experiment, respectively.

In the 1st year, during the productive cycle, temperatures were characterized by average maximum and minimum values of 28 °C and 16.6 °C, respectively. The maximum and minimum values were 44 °C and 11 °C, respectively. The maximum and minimum air humidity recorded during this period was 100% and 9%, respectively (with the maximum and minimum values being 90% and 38%, respectively). The 2nd year of the experimental period was characterized by average maximum and minimum temperatures of 27.7 °C and 17.9 °C (with maximum and minimum values of 35.6 °C and 15.3 °C, respectively). The maximum and minimum air humidity was 99% and 17%, respectively (with the average maximum and minimum values being 87.9% and 46.1%, respectively). During the 3rd productive cycle, the average maximum and minimum temperatures were 28.2 °C and 17.7 °C (with the maximum and minimum values being 37.7 °C and 4.8 °C, respectively). The maximum and minimum air humidity was 94% and 19%, respectively (with the average maximum and minimum values being 81.8% and 40.9%).

Field orography was characterized through images acquired with a UAV (Unmanned Aerial Vehicle) with altimetric measurement sensors, synchronized via GPS and processed in ArcGIS Pro, as described in [62].

Soil sampling (*n* = 28) was performed from the surface of the soil to a 30 cm depth (ca. 100 g), and then the samples were sieved (2.0 mm mesh to remove stones, coarse materials, and other debris), dried under 105 °C for 24 h, and subjected to 1 h of desiccation (until room temperature). Samples were then weighed to determine the dry mass and percentage of moisture. To determine the organic matter, the samples were heated to 550 °C for 4 h (i.e., until a constant weight), removed from the muffle at 100 °C, and then weighed (after desiccation until they reached room temperature for 1 h). Electrical conductivity and pH data were acquired by using a potentiometer, as described by [63]. Mineral quantification of the samples was carried out with an XRF analyzer (model XL3t 950 He GOLDD+, Niton Thermal Scientific, Munich, Germany) under a helium atmosphere, according to [64].

To assess the quality of the irrigation water during the last two years of the experimental period, we considered chemical (bicarbonate (HCO_3_^−^), sulfate (SO_4_^2−^), chloride (Cl^−^), phosphate (PO_4_^3−^), calcium (Ca^2+^), sodium (Na^+^), potassium (K^+^), and magnesium (Mg^2+^)) and physical (pH, temperature, and electrical conductivity) parameters. Alkalinity/bicarbonate was analyzed with titration in samples of 100 mL of water, using 0.1 N hydrochloric acid as the titrant, in the presence of 0.1% methyl orange [65]. Sulfate, chloride, and phosphate ions were quantified through photometry (Spectroquant NOVA 60, Merck, Darmstadt, Germany), using specific kits (1.14897, 1.14779, 1.14773, and 1.14842). Calcium, sodium, potassium, and magnesium ions were determined using a Metrohm (Model 761 Compact IC, Metrohom, Herisau, Switzerland) chromatograph, equipped with a column and pre-column (Metrosep cation 1–2, 6.1010.000), using an eluent mixture (4 mM tartaric acid/1 mM dipicolinic acid) at a flow rate of 1 mL/minute and a sample injection of 10 μL. Electrical conductivity (EC) and pH data were acquired using a Consort multiparameter analyzer (C 6030) and SP21 (pH) and SK20 T (CE) electrodes. Water classification in the vineyard considered dominant ions. The sodium adsorption index was determined and related to the electrical conductivity, in classes C and S [66]. Water data were projected in Piper and Wilcox diagrams with Grapher software (version 16.3.410).

To monitor the general physiological response of the vines to Zn enrichment, following agronomic practices in the vineyards, NDVI images were acquired during the experimental work period of the last two years (after the 4th and 1st foliar applications, respectively). Data were processed with an ArcGIS Pro [62].

In the vineyards, leaf gas exchange parameters of the vines were assessed on leaves (*n* = 4–6) from different plants (considering the 2nd youngest leaves fully expanded per treatment) [62]. Leaf rates of net photosynthesis (P_n_), stomatal conductance to water vapor (g_s_), and transpiration (E) were acquired under photosynthetic steady-state conditions after ca. 2 h of illumination (in the middle of the morning). A portable open-system infrared gas analyzer (Li-Cor 6400, Li-Cor, Lincoln, NE, USA) was used under environmental conditions, with external CO_2_ (ca. 400 ppm) and the photosynthetic photon flux density (PPFD) ranging between 1200 and 1400 µmol m^−2^ s^−1^. Leaf instantaneous water use efficiency (iWUE) was calculated as the Pn-to-E ratio, representing the units of assimilated CO_2_ per unit of water lost through transpiration [67].

Chlorophyll fluorescence *a* parameters were determined in each year of the experimental work on randomized leaves (*n* = 4–6) per treatment, using a fluorimeter PAM 2000 (H. Walz, Effeltrich, Germany), as described in [67,68], with some minor modifications. Briefly, the minimal fluorescence from the antennae (F_o_) and the maximal photochemical efficiency of the photosystem (PS) II (F_v_/F_m_) were acquired in overnight dark-adapted leaves, applying a low-irradiance red light (<0.5 μmol m^−2^ s^−1^) to obtain F_o_ and an actinic saturating light flash of ca. 7500 μmol m^−2^ s^−1^ to obtain the maximum fluorescence from the antennae (Fm), with the maximal photochemical efficiency of PSII (F_v_/F_m_) being estimated as ([(F_m_ − F_o_)/F_m_]). The other parameters were assessed under photosynthetic steady-state conditions, under natural irradiance (ca. 1000–1300 μmol m^−2^ s^−1^), with superimposed saturating light flashes, and included photochemical quenching, based on the concept of interconnected PSII antennae (q_L_); non-photochemical quenching (q_N_); and the actual PSII photochemical efficiency (F_v_′/F_m_′), as well as estimates of the following: the quantum yield of photosynthetic non-cyclic electron transport (Y(_II_)); the quantum yield of regulated energy dissipation of PSII (Y(_NPQ_)); and the non-regulated energy (heat and fluorescence) dissipation of PSII (Y(_NO_)) (with Y(_II_) + Y(_NPQ_) + Y(_NO_) = 1). All parameters and their meanings were determined using the formulas mentioned in [69,70,71,72,73].

At harvest, the Zn concentrations of randomized grapes (previously washed, dried at 60 °C until constant weight, and ground in an agate mortar) were determined after an acid digestion procedure with a mixture of HNO_3_^−^:HClO_4_ (4:1) followed by filtration [62]. Measurements (*n* = 3) were carried out using an atomic absorption spectrophotometer model, namely the Perkin Elmer AAnalyst 200 (Waltham, Massachusetts, MA, USA) fitted with a deuterium background corrector, and using the AA WinLab software program (Version 32).

The determination of total soluble solids (°Brix) was measured in the grape juice of randomized grapes for each treatment (*n* = 3) using a digital refractometer from Atago (Atago, Tokyo, Japan), and the values acquired were expressed as °Brix [74].

One-way and two-way ANOVA tests (*p* ≤ 0.05) were applied to statistically evaluate significant differences between treatments (a, b, c, d) and between assessment dates among the experimental periods of the three years (A, B), independently for each year. Mean comparisons were performed with Tukey’s test (95% confidence level).

## 5. Conclusions

Zinc enrichment of the *Vitis vinifera* Syrah winegrape variety can be induced with ZnO and ZnSO_4_ at concentrations of 150, 450, 900, and 1350 g.ha^−1^. Still, ZnO led to a higher accumulation of Zn, which makes this treatment the best one for Zn enrichment in winegrape production. In fact, with this treatment, a 2.3-fold increase can be reached at the maximum concentration (1350 g.ha^−1^). The threshold of toxicity was not reached through foliar application with either Zn treatment at any concentration, but potential benefits were found for photoassimilates synthesis and mobilization to the grapes during the final phase of the vine’s productive cycle, which determined a higher amount of total soluble solids (which favors winemaking), particularly with the higher concentrations of both Zn treatments.

## Figures and Tables

**Table 1:** Minimum, maximum, and average values ± S.D. of normalized difference vegetation index (NDVI) of the vineyard with *Vitis vinifera* cv. Syrah, during the experimental period (data collected in the 2nd and 3rd years, respectively, after the 4th and 1st foliar applications of ZnO or ZnSO_4_). Ctr = control samples.

Treatment Plots	NDVI					
	2nd Year			3rd Year		
	Minimum	Maximum	Average	Minimum	Maximum	Average
Ctr	0.14	0.85	0.66 ± 0.20	0.10	0.87	0.60 ± 0.21
ZnO (900 g.ha^−1^)	0.14	0.84	0.60 ± 0.21	-	-	-
ZnO (1350 g.ha^−1^)	0.15	0.85	0.61 ± 0.20	0.08	0.87	0.61 ± 0.22
ZnSO_4_ (900 g.ha^−1^)	0.19	0.85	0.65 ± 0.18	-	-	-
ZnSO_4_ (1350 g.ha^−1^)	0.13	0.85	0.55 ± 0.21	0.05	0.89	0.65 ± 0.21

**Table 2:** Average ± S.E. (*n* = 6–8) values of leaf gas exchange, net photosynthesis (P_n_), and stomatal conductance to water vapor (g_s_), as well as variation in the instantaneous water use efficiency (iWUE = P_n_/E), in leaves of *Vitis vinifera* of the variety Syrah during the experimental period (1st year after the 3rd foliar spray, 2nd year after the 4th foliar spray, and 3rd year after the 2nd and 4th foliar sprays with ZnO and ZnSO_4_). For all parameters, different letters indicate significant differences between the testing parameters for the different treatments (a, b, c), or between different assessments in the same treatment (A, B), independently for each year (statistical analysis using the two-way ANOVA test, *p* ≤ 0.05). Ctr = control samples.

		1st Year		2nd Year		3rd Year	
Sample		27 July	13 September	29 July	21 August	30 June	19 August
	P_n_ (µmol CO_2_ m^−2^ s^−1^)						
Ctr		17.5 ± 0.0 aA	13.6 ± 0.4 bB	16.2 ± 0.3 aA	9.8 ± 0.4 bB	13.0 ± 0.4 aA	9.1 ± 0.7 abB
	450	16.4 ± 0.1 abA	13.7 ± 0.1 bB	-	-	-	-
ZnO (g.ha^−1^)	900	15.1 ± 0.2 aB	15.3 ± 0.7 abA	16.2 ± 0.8 aA	12.0 ± 1.2 abB	-	-
	1350	-	-	16.5 ± 0.6 aA	14.0 ± 0.8 aA	12.0 ± 0.5 bA	7.2 ± 0.8 bB
	450	17.7 ± 0.5 abA	12.7 ± 0.5 bB	-	-	-	-
ZnSO_4_ (g.ha^−1^)	900	18.2 ± 0.2 aA	15.5 ± 0.3 aB	17.5 ± 0.4 aA	13.8 ± 0.5 abB	-	-
	1350	-	-	13.8 ± 0.8 aA	13.3 ± 0.5 aA	16.4 ± 0.2 aA	11.0 ± 0.7 aB
	g_s_ (mmol H_2_O m^−2^ s^−1^)						
Ctr		146.0 ± 1.7 aA	201.3 ± 5.8 bA	291.8 ± 61.8 aA	251.8 ± 50.3 aA	191.3 ± 9.1 aA	226.2 ± 17.8 aA
	450	131.0 ± 2.9 aB	197.8 ± 4.4 bA	-	-	-	-
ZnO (g.ha^−1^)	900	126.8 ± 5.2 aB	263.7 ± 39.9 aA	248.7 ± 50.2 aA	255.1 ± 57.7 aA	-	-
	1350	-	-	252.1 ± 43.7 aA	267.3 ± 39.9 aA	206.4 ± 14.9 aA	163.7 ± 27.1 aA
	450	151.0 ± 3.9 aA	167.3 ± 21.4 bA	-	-	-	-
ZnSO_4_ (g.ha^−1^)	900	150.7 ± 4.6 aA	201.2 ± 9.9 bA	248.7 ± 44.2 aA	213.8 ± 24.3 aA	-	-
	1350	-	-	187.0 ± 27.7 aA	263.1 ± 17.6 aA	251.6 ± 10.7 aA	219.5 ± 20.6 aA
		E (mmol H_2_O m^−2^ s^−1^)					
Ctr		4.5 ± 0.0 aA	5.4 ± 0.1 aB	3.4 ± 0.3 aA	2.7 ± 0.2 bA	3.48 ± 0.14 aB	5.39 ± 0.30 aA
	450	3.9 ± 0.0 abA	4.6 ± 0.1 bB	-	-	-	-
ZnO (g.ha^−1^)	900	3.7 ± 0.1 bA	4.3 ± 0.4 bA	3.3 ± 0.3 aA	3.2 ± 0.3 bA	-	-
	1350	-	-	3.5 ± 0.3 aA	3.9 ± 0.2 abA	3.91 ± 0.10 aA	4.27 ± 0.49 aA
	450	4.2 ± 0.1 abA	4.6 ± 0.3 bA	-	-	-	-
ZnSO_4_ (g.ha^−1^)	900	4.3 ± 0.1 abA	5.1 ± 0.1 aB	3.5 ± 0.3 aA	3.6 ± 0.2 abA	-	-
	1350	-	-	3.1 ± 0.2 aA	4.3 ± 0.2 aA	4.79 ± 0.11 aA	5.21 ± 0.32 aA
		iWUE (mmol CO_2_ mol^−1^ H_2_O)					
Ctr		3.9 ± 0.0 bA	2.5 ± 0.0 cB	5.0 ± 0.4 aA	3.7 ± 0.2 aB	3.91 ± 0.17 aA	1.67 ± 0.09 bB
	450	4.2 ± 0.1 aA	3.0 ± 0.0 bB	-	-	-	-
ZnO (g.ha^−1^)	900	4.2 ± 0.1 aA	3.6 ± 0.2 aB	5.3 ± 0.5 aA	3.8 ± 0.1 aB	-	-
	1350	-	-	4.8 ± 0.2 aA	3.7 ± 0.1 aB	2.64 ± 0.11 bA	1.67 ± 0.04 bB
	450	4.2 ± 0.1 aA	2.8 ± 0.1 bcB	-	-	-	-
ZnSO_4_ (g.ha^−1^)	900	4.2 ± 0.1 aA	3.1 ± 0.0 bB	5.1 ± 0.3 aA	3.8 ± 0.1 aB	-	-
	1350	-	-	4.6 ± 0.3 aA	3.1 ± 0.1 aB	3.55 ± 0.02 aA	2.11 ± 0.05 aB

**Table 3:** Average value ± S.E. (*n* = 6–8) of chlorophyll *a* fluorescence, maximal photochemical efficiency of PSII (F_v_/F_m_), estimate of the quantum yield of photosynthetic non-cyclic electron transport (Y(_II_)), estimate of the quantum yield of regulated energy dissipation (Y(_NPQ_)) and non-regulated energy (heat and fluorescence) dissipation (Y(_NO_)) of PSII, coefficient of non-photochemical (q_N_) and photochemical (q_L_) fluorescence quenching, and actual PSII efficiency of energy conversion under light (F_v_′/F_m_′) in leaves of *Vitis vinifera* L. variety Syrah during the experimental period (1st year after the 3rd foliar spray; 2nd year after the 4th foliar spray; and 3rd year after the 2nd and 4th foliar sprays, with ZnO and ZnSO_4_). For all parameters, different letters indicate differences between testing parameters for the different treatments (a, b), or between different assessments in the same treatment (A, B), independently for each year (statistical analysis using the two-way ANOVA test, *p* ≤ 0.05). Ctr = control samples.

		1st Year		2nd Year		3rd Year	
Sample		27 July	13 September	29 July	21 August	30 June	19 August
	F_v_/F_m_						
Ctr		0.770 ± 0.003 aA	0.806 ± 0.004 aA	0.773 ± 0.012 aA	0.755 ± 0.014 aA	0.767 ± 0.013 abA	0.786 ± 0.012 aA
	450	0.791 ± 0.004 aA	0.798 ± 0.005 aA	-	-	-	-
ZnO (g.ha^−1^)	900	0.767 ± 0.007 aA	0.780 ± 0.013 aA	0.738 ± 0.008 aA	0.750 ± 0.017 aA	-	-
	1350	-	-	0.728 ± 0.006 aA	0.720 ± 0.016 aA	0.776 ± 0.014 aA	0.808 ± 0.004 aA
	450	0.796 ± 0.007 aA	0.803 ± 0.010 aA	-	-	-	-
ZnSO_4_ (g.ha^−1^)	900	0.787 ± 0.011 aA	0.792 ± 0.007 aA	0.765 ± 0.015 aA	0.746 ± 0.007 aA	-	-
	1350	-	-	0.753 ± 0.018 aA	0.707 ± 0.019 aA	0.727 ± 0.016 bB	0.779 ± 0.005 aA
		F_v_′/F_m_′					
Ctr		0.451 ± 0.031 aA	0.486 ± 0.032 aA	0.467 ± 0.025 aA	0.440 ± 0.012 aA	0.403 ± 0.030 aA	0.512 ± 0.032 aA
	450	0.446 ± 0.027 aA	0.453 ± 0.036 aA	-	-	-	-
ZnO (g.ha^−1^)	900	0.557 ± 0.035 aA	0.488 ± 0.013 aA	0.500 ± 0.009 aA	0.451 ± 0.017 aA	-	-
	1350	-	-	0.486 ± 0.037 aA	0.441 ± 0.032 aA	0.469 ± 0.032 aA	0.552 ± 0.016 aA
	450	0.535 ± 0.027 aA	0.516 ± 0.025 aA	-	-	-	-
ZnSO_4_ (g.ha^−1^)	900	0.450 ± 0.030 aA	0.472 ± 0.019 aA	0.488 ± 0.045 aA	0.479 ± 0.035 aA	-	-
	1350	-	-	0.446 ± 0.017 aA	0.510 ± 0.018 aA	0.421 ± 0.035 aA	0.493 ± 0.016 aA
		Y_(II)_					
Ctr		0.358 ± 0.033 abA	0.325 ± 0.010 aA	0.338 ± 0.027 aA	0.239 ± 0.020 bA	0.265 ± 0.019 aA	0.314 ± 0.022 aB
	450	0.295 ± 0.020 bA	0.248 ± 0.027 aA	-	-	-	-
ZnO (g.ha^−1^)	900	0.412 ± 0.028 aA	0.302 ± 0.032 aA	0.306 ± 0.005 aA	0.232 ± 0.021 bA	-	-
	1350	-	-	0.300 ± 0.034 aA	0.273 ± 0.029 abA	0.289 ± 0.031 aA	0.334 ± 0.030 aA
	450	0.400 ± 0.018 aA	0.274 ± 0.030 aA	-	-	-	-
ZnSO_4_ (g.ha^−1^)	900	0.268 ± 0.021 bA	0.253 ± 0.024 aA	0.327 ± 0.032 aA	0.294 ± 0.023 abA	-	-
	1350	-	-	0.310 ± 0.016 aA	0.371 ± 0.032 aA	0.267 ± 0.042 aA	0.332 ± 0.020 aA
		Y_(NPQ)_					
Ctr		0.442 ± 0.039 abA	0.483 ± 0.026 aA	0.450 ± 0.016 aA	0.558 ± 0.014 aA	0.538 ± 0.025 aA	0.373 ± 0.037 aB
	450	0.546 ± 0.019 aA	0.520 ± 0.034 aA	-	-	-	-
ZnO (g.ha^−1^)	900	0.403 ± 0.033 bA	0.478 ± 0.028 aA	0.474 ± 0.025 aA	0.572 ± 0.022 abA	-	-
	1350	-	-	0.499 ± 0.041 aA	0.528 ± 0.042 abA	0.508 ± 0.036 aA	0.348 ± 0.015 aB
	450	0.401 ± 0.026 bA	0.503 ± 0.028 aA	-	-	-	-
ZnSO_4_ (g.ha^−1^)	900	0.512 ± 0.023 abA	0.522 ± 0.023 aA	0.505 ± 0.042 aA	0.500 ± 0.034 abA	-	-
	1350	-	-	0.524 ± 0.017 aA	0.429 ± 0.035 bA	0.519 ± 0.049 aA	0.380 ± 0.023 aB
		Y_(NO)_					
Ctr		0.199 ± 0.009 aA	0.193 ± 0.018 aA	0.212 ± 0.016 aA	0.203 ± 0.007 aA	0.196 ± 0.010 aB	0.313 ± 0.032 aA
	450	0.158 ± 0.009 aA	0.232 ± 0.014 aA	-	-	-	-
ZnO (g.ha^−1^)	900	0.184 ± 0.016 aA	0.221 ± 0.008 aA	0.219 ± 0.024 aA	0.196 ± 0.009 aA	-	-
	1350	-	-	0.202 ± 0.012 aA	0.199 ± 0.016 aA	0.204 ± 0.015 aB	0.318 ± 0.028 aA
	450	0.199 ± 0.013 aA	0.223 ± 0.012 aA	-	-	-	-
ZnSO_4_ (g.ha^−1^)	900	0.214 ± 0.014 aA	0.225 ± 0.006 aA	0.168 ± 0.014 aA	0.207 ± 0.011 aA	-	-
	1350	-	-	0.167 ± 0.011 aA	0.200 ± 0.008 aA	0.214 ± 0.017 aA	0.288 ± 0.015 aA
		q_N_					
Ctr		0.809 ± 0.031 aA	0.823 ± 0.030 aA	0.807 ± 0.019 aA	0.845 ± 0.004 aA	0.856 ± 0.018 aA	0.696 ± 0.049 aB
	450	0.873 ± 0.012 aA	0.819 ± 0.027 aA	-	-	-	-
ZnO (g.ha^−1^)	900	0.768 ± 0.035 aA	0.801 ± 0.012 aA	0.785 ± 0.028 aA	0.845 ± 0.012 aA	-	-
	1350	-	-	0.800 ± 0.031 aA	0.823 ± 0.031 aA	0.822 ± 0.026 aA	0.674 ± 0.025 aB
	450	0.772 ± 0.028 aA	0.801 ± 0.021 aA	-	-	-	-
ZnSO_4_ (g.ha^−1^)	900	0.827 ± 0.020 aA	0.819 ± 0.013 aA	0.831 ± 0.035 aA	0.806 ± 0.032 aA	-	-
	1350	-	-	0.856 ± 0.014 aA	0.766 ± 0.024 aA	0.823 ± 0.033 aA	0.724 ± 0.023 aA
		q_L_					
Ctr		0.684 ± 0.049 aA	0.516 ± 0.056 aA	0.604 ± 0.107 aA	0.400 ± 0.036 abA	0.650 ± 0.032 aA	0.445 ± 0.053 aA
	450	0.538 ± 0.073 aA	0.403 ± 0.042 aA	-	-	-	-
ZnO (g.ha^−1^)	900	0.564 ± 0.055 aA	0.460 ± 0.058 aA	0.443 ± 0.018 aA	0.370 ± 0.040 bA	-	-
	1350	-	-	0.450 ± 0.039 aA	0.478 ± 0.044 abA	0.477 ± 0.075 aA	0.420 ± 0.060 aA
	450	0.586 ± 0.047 aA	0.358 ± 0.052 aA	-	-	-	-
ZnSO_4_ (g.ha^−1^)	900	0.478 ± 0.060 aA	0.379 ± 0.032 aA	0.513 ± 0.049 aA	0.453 ± 0.033 abA	-	-
	1350	-	-	0.564 ± 0.044 aA	0.574 ± 0.058 aA	0.506 ± 0.084 aA	0.514 ± 0.037 aA

**Table 4:** Average value ± S.E. (*n* = 3) of Zn concentration in grapes of *Vitis vinifera* variety Syrah at harvest during the experimental period (1st, 2nd, and 3rd years). Letters a and b indicate significant differences among treatments (statistical analysis using the single-factor ANOVA test, *p* ≤ 0.05). Ctr = control samples.

Treatment	Cv. Syrah Zn Content (mg.kg^−1^)		
	1st Year	2nd Year	3rd Year
Ctr	7.94 a	4.51 b	10.15 b
ZnO (150 g.ha^−1^)	11.07 a	-	-
ZnO (450 g.ha^−1^)	12.31 a	-	-
ZnO (900 g.ha^−1^)	11.30 a	7.35 ab	-
ZnO (1350 g.ha^−1^)	-	10.37 a	11.89 a
ZnSO_4_ (150 g.ha^−1^)	9.36 a	-	-
ZnSO_4_ (450 g.ha^−1^)	12.26 a	-	-
ZnSO_4_ (900 g.ha^−1^)	10.59 a	6.08 ab	-
ZnSO_4_ (1350 g.ha^−1^)	-	8.25 ab	10.72 b

## Data Availability

Data are contained within the article.
